# Monitoring of enteropathogenic Gram-negative bacteria in wastewater treatment plants: a multimethod approach

**DOI:** 10.1007/s11356-024-33675-2

**Published:** 2024-05-20

**Authors:** Agata Stobnicka-Kupiec, Małgorzata Gołofit-Szymczak, Marcin Cyprowski, Rafał L. Górny

**Affiliations:** Central Institute for Labour Protection—National Research Institute, Czerniakowska Street 16, Warsaw, Poland

**Keywords:** Enteropathogenic bacteria, Occupational exposure, Wastewater treatment plant, Multiplex PCR

## Abstract

The wastewater treatment processes are associated with the emission of microbial aerosols, including enteropathogenic bacteria. Their presence in this work environment poses a real threat to the health of employees, both through the possibility of direct inhalation of the contaminated air and indirectly through the pollution of all types of surfaces with such bioaerosol particles. This study aimed to investigate the prevalence of enteropathogenic bacteria in the air, on surfaces, and in wastewater samples collected in four wastewater treatment plants (WWTPs). The effectiveness of conventional culture-biochemical, as well as spectrometric and molecular methods for the rapid detection of enteropathogenic bacteria at workstations related to particular stages of wastewater processing, was also evaluated. Bioaerosol, surface swab, and influent and effluent samples were collected from wastewater plants employing mechanical–biological treatment technologies. The air samples were collected using MAS-100 NT impactor placed at a height of 1.5 m above the floor or ground, simulating aspiration from the human breathing zone. Surface samples were collected with sterile swabs from different surfaces (valves, handles, handrails, and coveyor belts) at workplaces. The raw influent and treated effluent wastewater samples were aseptically collected using sterile bottles. The identification of bacterial entheropathogens was simultaneously conducted using a culture-based method supplemented with biochemical (API) tests, mass-spectrometry (MALDI TOF MS), and molecular (multiplex real-time PCR) methods. This study confirmed the common presence of bacterial pathogens (including enteropathogenic and enterotoxigenic *Escherichia coli*, *Salmonella* spp., *Campylobacter* spp., and *Yersinia enterocolitica*) in all air, surface, and wastewater samples at studied workplaces. Higher concentrations of enteropathogenic bacteria were observed in the air and on surfaces at workplaces where treatment processes were not hermetized. The results of this study underline that identification of enteropathogenic bacteria in WWTPs is of great importance for the correct risk assessment at workplaces. From the analytical point of view, the control of enteropathogenic bacterial air and surface pollution using rapid multiplex-PCR method should be routinely performed as a part of hygienic quality assessment in WWTPs.

## Introduction

Wastewater treatment processes are widely recognized as significant sources of microbial aerosols, posing potential health risks for wastewater treatment plant (WWTP) workers (Grisoli et al. 2009; Heinonen-Tanski et al. 2009; Wu et al. 2019). Enteropathogenic bacteria (EB) are known to pose a global health threat, and wastewater is their major natural reservoir (Jia and Zhang 2020). Typically transmitted through the fecal–oral route, EB enter WWTPs via sewage containing excreted feces, contributing to millions of Gram-negative bacteria per milliliter and including various pathogenic strains like *Salmonella* spp., *Shigella* spp., *Escherichia coli*, *Yersinia enterocolitica*, and *Campylobacter* spp. (Jia and Zhang 2020; Chahal et al. 2016; Ørmen et al. 2019).

The wastewater entering the treatment plant consists of a mixture of domestic and industrial wastewater, for example from animal farms or hospitals, as well as rainwater, containing a variety of microorganisms. Enteropathogens originate directly or indirectly from human and animal excreta, especially from the feces of sick individuals or carriers. It is important to note that many enteropathogens are released in large quantities with the feces not only during infections but also several days or weeks before the onset of symptoms, as well as after the disease has ceased. According to the US Environmental Protection Agency (EPA Environmental Protection Agency 2018), when 1–10% of the population of a given area excrete pathogenic bacteria in the amount of 10^8^ CFU/g of feces, this results in the presence of these pathogens in sewage in the amount of 10^5^–10^7^ CFU/L. Moreover, wastewater treatment may not be sufficent for enteropathogenic bacteria removal and bacterial pathogens can survive in such an environment up to several months (Teklehaimanot et al. 2015; Yi and Shane 2018). Based on the accessible data, in the workplace environment of wastewater treatment plants, the bacterial enteropathogens identified so far are as follows: *Salmonella* (*S*. Paratyphi, *S.* Enteritidis, *S.* Typhimurium), *Shigella* (*Sh. dysenteriae*), *Vibrio* (*V. cholerae*, *V. parahaemolyticus*, *V. vulnificus*, *V. fluvialis*), *Campylobacter* (*C. jejuni*, *C. lari*, *C. coli*), *Escherichia* (*E. coli*), and *Yersinia* (*Y. enterocolitica*, *Y. pseudotuberculosis*) (Anastasi et al. 2012; Langeland 1982; Jones 2001).

Research reports from recent years show that the issue of pathogenic bacteria in bioaerosols in wastewater treatment plants remains a relevant problem (Jabeen et al. 2023). Wastewater treatment processes generate aerosols of different sizes, carrying biological agents present in wastewater, which can be subsequently deposited on surfaces (Han et al. 2013). Consequently, WWTP employees may be exposed to bioaerosol pathogens during their working activities, either through inhalation/deglutition or direct contact with contaminated surfaces, clothing, tools, or hands (Muzaini et al. 2021). Studies indicate that WWTP workers often report acute non-specific gastrointestinal symptoms that may result from contact with enteropathogenic bacteria (Albatanony and El-Shafie 2011; Jeggli et al. 2004; Friis et al. 1998).

Given these concerns, an important part of safety work management is proper health risk assessment. Identifying pathogens in the occupational environment is crucial in the initial stages of risk assessment. Traditionally, detection and identification of pathogenic bacteria rely on various classic diagnostic tools such as microscopy, cultivation on microbiological media, and biochemical tests. The results obtained using these traditional methods, however, often require confirmation with spectrometric or molecular methods (Suzuki et al. 2018; Ørmen and Madslien 2018).

This study aims to investigate the prevalence of enteropathogenic Gram-negative bacteria (*Escherichia coli*, *Salmonella* spp, *Shigella* spp., *Yersinia enterocolitica*, *Campylobacter* spp.) in bioaerosol and surface swab samples collected in WWTPs, as well as in raw influent and treated effluent samples. Additionally, the study evaluates the suitability of combined culture-biochemical (API), spectrometric (MALDI TOF MS), and molecular (multiplex real-time PCR) method for rapid detection of enteropathogenic bacteria in the WWTP environment and quick evaluation of efficacy of wastewater treatment processes.

## Methodology

### Sampling sites

All studied WWTPs were located in central Poland and were categorized as very small, small, medium, and large facilities depending on their capacity. All the examined wastewater treatment plants based on mechanical–biological wastewater treatment involve sequencing batch reactors and plate-and-frame filter press and were purifying municipal wastewater.

Table 1 provides description of the examined WWTPs, including their detailed characteristics. All samples were collected by the authors of this study after obtaining the necessary permits issued by the authorities of all studied WWTPs.

**Table 1:** Description of wastewater treatment plants (WWTPs) and their workplaces

Size of WWTP	Very small		Small		Medium		Large	
Workplace	Capacity of WWTP [m^3^/day]							
	< 1500		10,000		60,000		> 300,000	
	H^*)^	*n*	H	*n*	H	*n*	H	*n*
Wastewater pumping section	−	6	−	4	−	4	+	3
Screens section	+ / −	2	−	4	+ / −	3	+	3
Grit chamber	+	2	+	2	−	3	+	3
Bioreactor	+	2	+	2	+	2	−	3
Dewatering sludge section	−	5	+	2	+	2	+	2
Thickening sludge section	+	2	+ / −	6	+	3	+	2

^*)^ H—process hermetization; + yes, − no, + / − partially; *n*—number of samples

### Bioaerosol sampling

**Fig. 1:**
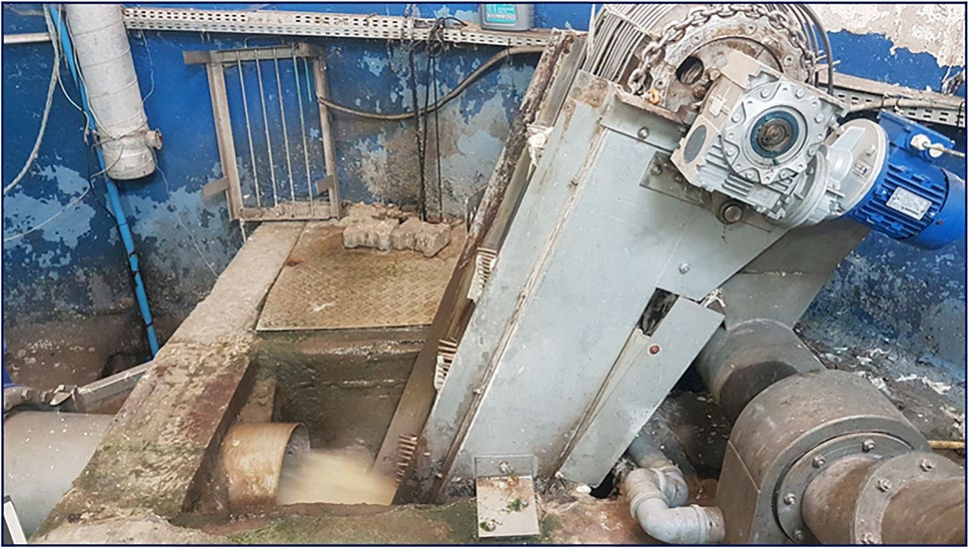
An example of not hermitized process at wastewater pumping section in one of the studied WWTP

In total, 72 bioaerosol samples (32 collected from hermetized area and 40 gathered in not or partially hermetized areas—see Fig. 1), were collected during regular working hours from various workplaces within WWTPs including: wastewater pumping Sect. (17), screens Sect. (12), grit chamber (10), bioreactor (9), dewatering sludge Sect. (11), and thickening sludge Sect. (13). The air samples (volume of each sample = 100 L; flow rate 100L/min) were collected using a single-stage MAS impactor (model 100-NT, MBV AG, Stäfa, Switzerland) on trypticase soy agar (TSA) with 5% defibrinated sheep blood (bioMérieux, Marcy l’Étoile, France). All bioaerosol samples were collected at a height of 1.5 m above the floor or ground, simulating aspiration from the human breathing zone (EN 13098:^2019^). Throughout the bioaerosol measurements, the temperature and relative humidity of the air were monitored using a portable thermo-hygrometer (model Testo 410–2, Testo SE & Co. KGaA, Titisee-Neustadt, Germany). To assess the potential influence of external sources of microbiological pollution on air quality at the studied workplaces, background bioaerosol samples (atmospheric air) were simultaneously collected in close vicinity of the studied facility (upwind, approximately 300 m from the border of each examined WWTP).

### Surface swab sampling

In total, 51 surface samples were collected using sterile swabs prewetted with Amies transport medium (DeltaSwab, Spain) from various surfaces within selected workplaces, including machine valves, machine handles, conveyor belts, and handrails (Table 2). After shaking the swabs for 10 min at room temperature, a series of dilutions of each sample (10^−1^ to 10^−5^) were prepared. Triplicates of 0.1 mL from each dilution were then inoculated onto trypticase soy agar (TSA) supplemented with 5% defibrinated sheep blood (bioMérieux) for further bacterial analysis.

**Table 2:** Surface swabs collection points

Workplace	Type of surface	*n*	
		H	NH
Wastewater pumping section	Machine valve, machine handle	7	3
Screens section	Machine handle, handrail, conveyor belts	4	4
Grit chamber	Machine handle, handrail	3	7
Bioreactor	Machine handle, handrail	3	6
Dewatering sludge section	Machine handle, handrail	4	3
Thickening sludge section	Machine handle, handrail	3	4

*n*—number of samples; H—hermitized process; NH—not hermetized process

### Wastewater samples collection

A total of 16 raw wastewater influent and 16 treated wastewater effluent samples were aseptically collected using 1-L sterile bottles. Prior to sample collection, the sampling bottles were thoroughly rinsed with distilled water and autoclaved at 121 °C for 15 min. A series of dilutions for each sample (10^−1^ to 10^−9^) was prepared and used in triplicate, with 0.1 mL of each dilution inoculated onto the appropriate medium.

### Laboratory analyses

Agar plates with bioaerosol, surface swab, and wastewater samples were incubated at 37 °C for 24 h in aerobic conditions for *E. coli, Salmonella* spp., *Shigella* spp., *Y. enterocolitica*, and at 37 °C for 24 h in microaerophilic conditions for *Campylobacter* spp., as specified by the manufacturer’s instructions using GenBag Microaer (bioMérieux). After incubation, all colonies were counted. The total concentration of bacteria in bioaerosol was determined as colony-forming units per cubic meter of the air (CFU/m^3^), on surfaces as CFU per square centimeter (CFU/cm^2^), while in untreated and treated wastewater samples as CFU per liter (CFU/L). All isolated bacterial colonies were morphologically evaluated and classified into Gram-positive/Gram-negative groups based on Gram staining results.

**Fig. 2:**
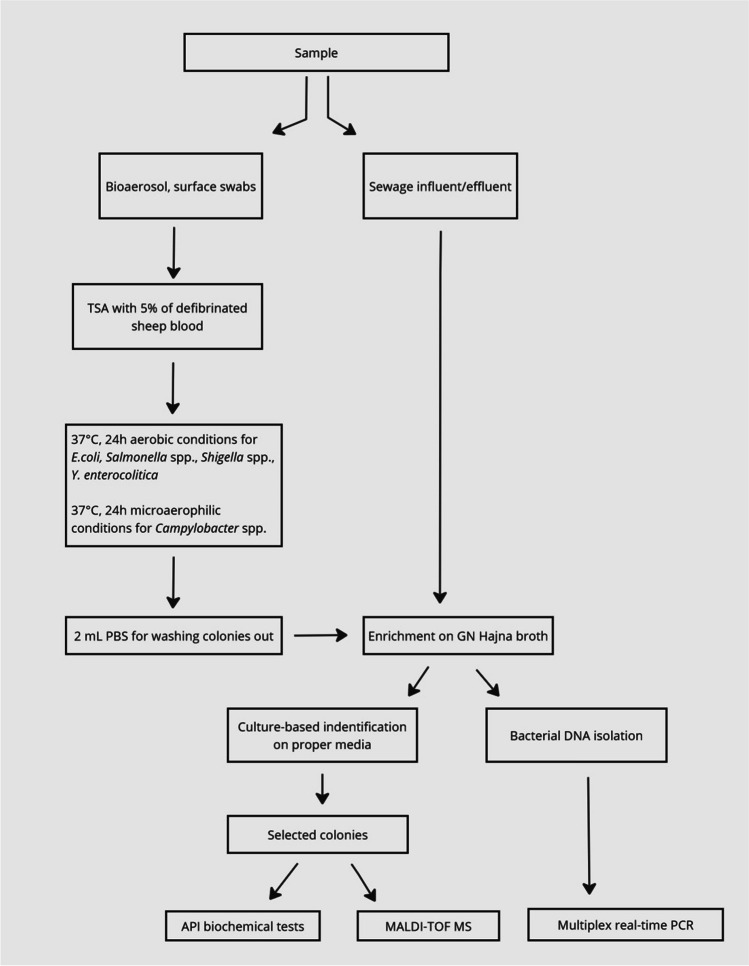
General scheme of the study

The grown bacterial colonies were then washed with 2 mL of PBS buffer and intended for inoculation into GN Hajna broth (Merck Eurolab GmbH, Germany) for sample enrichment. Subsequently, the identification of enteropathogenic bacteria was simultaneously carried out using a culture-based, biochemical (API), spectrometric (MALDI TOF MS), and molecular (multiplex real-time PCR) methods. The general scheme of all performed analytical steps is presented in Fig. 2.

### Culture-based isolation of Escherichia coli, Salmonella spp., Yersinia enterocolitica, Shigella spp., and Campylobacter spp.

The isolation of *E. coli* (a), *Salmonella* spp. (b), *Shigella* spp. (c), *Y. enterocolitica* (d), and *Campylobacter* spp. (e) involved enrichment techniques in GN Hajna broth following standard methods (APHA 2001). After enriching the culture for 6 to 8 h at 37 °C (a–c), 25 °C (d), and 41 °C (e), 1 mL of the culture or its subsequent serial dilutions were streak-plated on various selective media: xylose lysine deoxycholate (XLD), Hektoen agar, MacConkey agar (with crystal violet, sodium chloride, and 0.15% bile salts), *Yersinia* CIN agar, and CASA agar (all media: Merck Eurolab GmbH). The plates were then incubated under aerobic and microaerophilic (for CASA agar only) conditions for 24 h at 37 °C, 25 °C, and 41 °C to detect and enumerate expected colonies. Moreover, for each plate, three to five presumptive colonies of the target bacteria were selected and sub-cultured twice on brain heart infusion agar, followed by plating on nutrient agar (Merck), prior to further identification.

### Bacterial identification

#### Biochemical method (API tests)

All isolated microbial colonies were identified to the genus and/or species level, considering their macroscopic and microscopic features. The identification process included: observation of motility, Gram staining, and oxidase and catalase activities of the isolates. Bacterial identification was further complemented with appropriate biochemical API tests, specifically ‘10S’ or ‘20E’ for *Enterobacteriaceae* and ‘Campy’ for *Campylobacter* spp. (bioMérieux). The final confirmation of taxonomical identification was accomplished using the APIweb database (bioMérieux).

#### Spectrometric method (MALDI TOF MS)

The identification of isolated bacterial strains was also conducted using matrix-assisted laser desorption/ionization time-of-flight (MALDI-TOF) mass spectrometry (MALDI Biotyper, Bruker Daltonik, Germany). In brief, bacterial colonies (after 18–24 h incubation) were isolated from the TSA agar medium (Merck Eurolab GmbH). Cell proteins were extracted with ethanol, followed by a mixture of formic acid and acetonitrile. After drying 1 μL of supernatant samples on a metal plate and adding a matrix solution (1 μL), the plate with the samples was placed in the MALDI Biotyper chamber for analysis. A score ≥ 2.0 indicated high-confidence identification (Kozdrój et al. 2019).

### Molecular methods

#### Bacterial DNA isolation

The isolation of bacterial DNA from bacterial strains was performed using Syngen DNA Mini Kit (Syngen, Wroclaw, Poland), while DNA from bioaerosol, surface swab, and wastewater samples was extracted using Syngen Stool DNA Mini Kit (Syngen) following the manufacturer’s recommended procedure. The extracted DNA samples were stored at − 80 °C for further analysis.

#### Multiplex real-time PCR

Multiplex real-time polymerase chain reaction (multiplex-real-time PCR) was conducted using the CFX96 real-time PCR thermocycler (BioRad, USA). The detection of *E. coli* and *Shigella* spp. (EHEC—enterohemorrhagic *E. coli*, STEC—Shiga toxin-producing *E. coli*, EPEC—enteropathogenic *E. coli*, ETEC—enterotoxigenic *E. coli*, EIEC—enteroinvasive *E. coli*, *Shigella* spp., *Shigella dysenteriae* type 1), as well as *Salmonella* spp., *Campylobacter* spp., and *Y*. *enterocolitica*, was carried out using VIASURE *E. coli* Typing Real-Time PCR Detection Kit, as well as VIASURE *Salmonella*, *Campylobacter* & *Y. enterocolitica* Real-Time PCR Detection Kit (both: CerTest Biotec, Spain), respectively.

The target genes utilized for rapid detection and identification of *Salmonella*, *Campylobacter*, and *Y. enterocolitica* in multiplex real-time PCR assays were the virulence invA gene (*Salmonella* invasion protein gene), 16SrRNA gene, and ail (attachment-invasion locus) gene, respectively. For identification of colipathotypes and *Shigella* spp., the virulence stx1/stx2 and eae genes were used for EHEC, stx1/stx2 genes were used for STEC, eae gene was used for EPEC, lt and st1a/st1b genes were used for ETEC, ipaH gene were used for EIEC/*Shigella* spp., and stx1/stx2 and ipaH genes were used for *Shigella dysenteriae* type 1.

All laboratory activities were conducted following the manufacturer’s instructions. In brief, each multiplex reaction mixture (20 µL) contained 15 µL of master mix of specific primers/probes, dNTPS, buffer, polymerase, retrotranscriptase in a stabilized format, and an internal control to discard the inhibition of the polymerase activity. Additionally, 5 µL of the DNA sample was included in each reaction. The mixtures underwent the following conditions: initial denaturation at 95 °C for 2 min, followed by 49 cycles of denaturation at 95 °C for 10 s and annealing/extension at 60 °C (50 s). As per the manufacturer’s procedure, fluorogenic data were collected through the FAM, Cy5, ROX, and HEX channels. Each run included both negative and positive controls (CerTest Biotec). To minimize potential contamination, all analytical steps were conducted in separate rooms.

### Statistical analysis

As all independent variables were not normally distributed (based on Shapiro–Wilk test), the obtained results were statistically analyzed with Kruskal–Wallis, Mann–Whitney *U*, Chi-squared, and Fisher exact tests, as well as Spearman rank correlation coefficient using STATISTICA data analysis software system, version 10. (StatSoft Inc., Tulsa, USA). *p* values below 0.05 were considered statistically significant.

## Results

### Quantitative and qualitative analysis of bioaerosol samples

The concentrations of bacterial aerosols in WWTPs are presented in Table 3. The average bacterial concentrations in the air ranged from 98 to 18,850 CFU/m^3^, 120 to 24,280 CFU/m^3^, 215 to 17,350 CFU/m^3^, and 245 to 4980 CFU/m^3^ in very small, small, medium, and large WWTPs, respectively. Bacterial aerosol concentrations at workplaces in WWTPs were significantly higher than those measured in the atmospheric background near the studied facilities (Mann–Whitney *U* test: *p* = 0.004). Moreover, statistically significant differences between the studied workplaces were also observed (Kruskal–Wallis test: *p* = 0.001). The highest concentrations of bacterial aerosols were detected within the wastewater pumping section in very small and small WWTPs, at the grit chamber section in medium WWTPs, and within the bioreactor area in the large facilities. Taking into account the hermetization of technological processes (Table 4), significantly higher concentrations of bacteria in the air were recorded at workplaces without the possibility of limiting the spread of this type of biological pollutants (Mann–Whitney *U* test: *p* = 0.0001).

**Table 3:** Concentrations of bacteria [CFU/m^3^] at the studied workplaces in WWTPs depending on their capacity

Workplace	Total bacteria concentration CFU/m^3^							
	WWTPs capacity [m^3^/day]							
	< 1500		10,000		60,000		> 300,000	
	Median	Range	Median	Range	Median	Range	Median	Range
Wastewater pumping section	18,850	15,850–18,850	24,280	20,280–26,280	13,850	1200–15,850	1990	1890–2100
Screens section	8165	6670–10,050	8820	6420–11,180	4743	1410–11,500	245	110–980
Grit chamber	150	140–153	325	240–380	17,350	15,850–18,850	465	370–530
Bioreactor	98	90–110	120	70–180	215	150–280	4980	3230–6860
Dewatering sludge section	13,225	11,890–14,560	190	160–230	690	550–820	520	470–590
Thickening sludge section	1505	1100–1860	265	180–1170	700	640–720	275	210–360
Backgorund (atmospheric air)	220	190–370	170	132–200	320	250–440	70	60–120

**Table 4:** Concentrations of bacteria [CFU/m^3^] at the studied workplaces in WWTPs depending on hermitization of the treatment processes

Workplace	Process hermetization			
	H		NH	
	Total bacteria concentration [CFU/m^3^]			
	Median	Range	Median	Range
Wastewater pumping section	1990	1890–2100	18,850	1370–26,280
Screens section	245	110–980	6575	320–11,180
Grit chamber	320	140–530	17,350	15,850–18,850
Bioreactor	108	90–280	4980	3230–6860
Dewatering sludge section	225	160–780	13,225	11,890–14,560
Thickening sludge section	730	180–1170	4955	1430–8050

H—process with hermetization; NH—process without hermetization/partially hermetized

Within the tested processing areas, the air temperature ranged between 22.3 and 25.8 °C, while relative humidity ranged between 52.7 and 60.3%. Neither relative air humidity nor temperature significantly influenced bacterial aerosol concentrations (Spearman rank correlation coefficient test: *p* > 0.05).

**Fig. 3:**
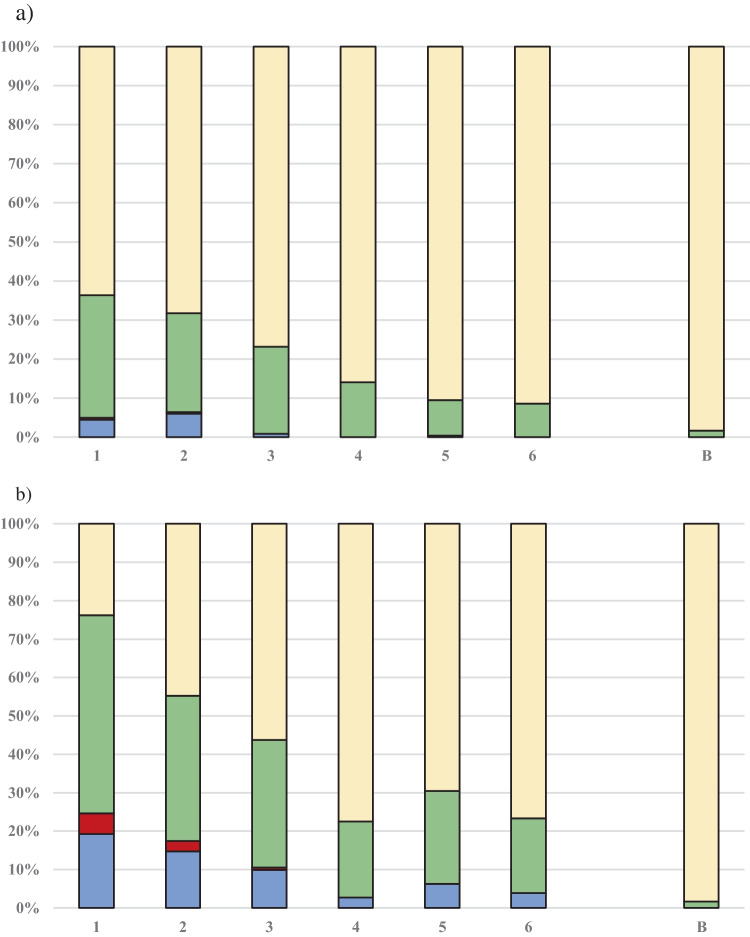
Percentage contribution of bacterial groups to total bacterial microbiota isolated from the air at hermitized (**a**) and not or partially hermitized (**b**) workplaces in WWTPs, as well as in background air (**c**). Notes: *Escherichia coli*, *Salmonella* spp.,  other non-enteropathogenic Gram-negative rods,  Gram-positive bacteria; 1—wastewater pumping section; 2—screens section, 3—grit chamber, 4—bioreactor, 5—dewatering sludge section, 6—thickening sludge section, B—background

The percentage distributions of bacterial groups identified in bioaerosols at workplaces within the studied WWTPs and in atmospheric air are illustrated in Fig. 3. In bioaerosols collected at workplaces where technological processes were hermetized, Gram-positive bacteria were the predominant group of microorganisms (forming 63.7–91.4% of the total studied microbiota), followed by non-enteropathogenic Gram-negative rods (8.6–31.4%). *E. coli* constituted a maximum of 6% of the total microbiota, while *Salmonella* spp. did not exceed 0.5% and was only detected in the initial steps of the technological processes (wastewater pumping and screen sections). Similar picture was observed at not hermitized workplaces where Gram-positive bacteria were the predominant group of microorganisms (forming 23.8–77.5% of the total studied microbiota), followed by non-enteropathogenic Gram-negative rods (19.4–51.6%) and *E. coli* (2.7–19.2%). *Salmonella* accounted for 5.4% in the wastewater pumping area, 2.7% in the screens section, and 0.6% in the grit chamber. In atmospheric air, Gram-positive bacteria were the most abundant group of isolated microorganisms, constituting 98.4% of the total isolated bacteria.

All enteropathogenic bacterial strains isolated from bioaerosol samples are listed in Table 5. Enteropathogenic strains of *E. coli* (EPEC), *E. coli* (ETEC), and *Salmonella* spp. were detected in bioaerosol samples collected in wastewater pumping and screen sections. They were significantly more often isolated from bioaerosol samples collected at workplaces without hermitization of technological processes (Chi-square test: *p* = 0.0000, Fisher’s exact test: *p* = 0.0000).

**Table 5:** Enteropathogenic bacteria identified among tested bioaerosol samples with biochemical, spetrometric, and molecular methods

Identification method	Biochemical (API)	Spectrometric (MALDI TOF MS)	Molecular (multiplex real-time PCR)		
Workplace	Species/genus			Total positive samples	
				NH	H
Wastewater pumping section	*E. coli*	*E. coli*	*E. coli* (EPEC)	5/14	1/3
	*E. coli*	*E. coli*	*E. coli* (ETEC)	2/14	0/3
	*E. coli*	*E. coli*	*E. coli* (OTC)	11/14	1/3
	*Salmonella* spp.	*S. enterica* ssp*. arizonae*	*Salmonella* spp.	1/14	0/3
	*Salmonella* spp.	*S. enterica* ssp*. enterica* ser. Hadar	*Salmonella* spp.	1/14	0/3
	No identification	No identification	*Salmonella* spp.	2/14	0/3
Screens section	*E. coli*	*E. coli*	*E. coli* (EPEC)	7/9	1/3
	*E. coli*	*E. coli*	*E. coli* (EPEC)	1/9	0/3
	*E. coli*	*E. coli*	*E. coli* (OTC)	6/9	2/3
	*Salmonella* spp.	*Salmonella* spp.	*Salmonella* spp.	1/9	0/3
Grit chamber	*E. coli*	*E. coli*	*E. coli* (OTC)	1/3	0/7
Bioreactor	*E. coli*	*E. coli*	*E. coli* (OTC)	1/3	2/6
Dewatering sludge section	*E. coli*	*E. coli*	*E. coli* (OTC)	1/5	3/6
Thickening sludge section	*E. coli*	*E. coli*	*E. coli* (OTC)	2/6	2/7
Backgorund	ND	ND	ND	-	-

EPEC—enteropathogenic *E. coli*, ETEC—enterotoxigenic *E. coli*, OTC—other than colipathotypes; ND—not detected; H—process with hermetization; NH—process without hermetization/partially hermetized

### Quantitative and qualitative analysis of surface swab samples

Concentrations of bacteria on surfaces in WWTPs ranged between 2.2 and 27.2 CFU/cm^2^ at hermatized workplaces (H) and between 8.9 and 110.8 CFU/cm^2^ at not or partially hermetized workplaces (NH) (Table 6). The highest concentrations of bacteria on both H and NH surfaces were observed within the screen Sect. (20.7 CFU/cm^2^ and 65.1 CFU/cm^2^, respectively), followed by surfaces in the wastewater pumping section area (15.3 CFU/cm^2^ and 52.3 CFU/cm^2^, respectively).

**Table 6:** Concentrations of bacteria on surfaces [CFU/cm^2^] at the studied workplaces in WWTPs depending on process hermetization

Workplace	Process hermetization			
	H		NH	
	Total bacteria concentration CFU/cm^2^			
	Median	Range	Median	Range
Wastewater pumping section	15.3	9.1–23.4	52.3	19.1–75.4
Screens section	20.7	17.5–27.2	65.1	17.2–110.8
Grit chamber	5.9	3.1–8.7	24.9	9.3–36.1
Bioreactor	6.3	2.8–8.1	16.1	8.9–21.5
Dewatering sludge section	11.9	6.7–17.4	22.9	16.5–29.2
Thickening sludge section	6.2	2.2–9.7	14.3	13.4–15.1

*CFU* colony forming units

**Fig. 4:**
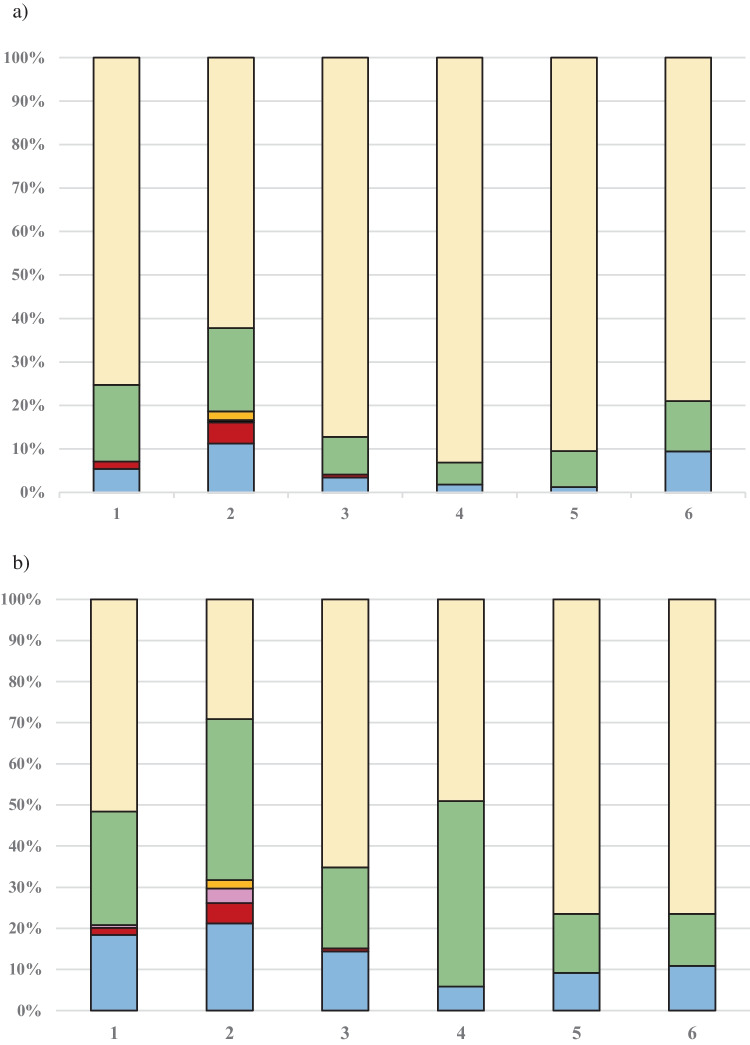
Percentage contribution of bacterial groups to total bacterial microbiota isolated from the surfaces at hermitized (**a**) and not or partially hermitized (**b**) workplaces in WWTPs, as well as in background air (**c**). Notes: *Escherichia coli*, *Salmonella* spp., *Yersinia* spp., *Campylobacter* spp.,  other non-enteropathogenic Gram-negative rods,  Gram-positive bacteria; 1—wastewater pumping section; 2—screens section, 3—grit chamber, 4—bioreactor, 5—dewatering sludge section, 6—thickening sludge section

The percentage distributions of bacterial groups identified on surfaces among the tested areas in the studied WWTPs are depicted in Fig. 4. On all studied surfaces at workplaces within WWTPs, i.e., with and without hermetization of technological processes, Gram-positive bacteria were the predominant group of microorganisms (forming 49.1–93.1% of the total microbiota), followed by non-enteropathogenic Gram-negative rods (5.1–45.1%). The only exception was observed in case of surfaces in not hermetized screens section. In this case, Gram-negative bacteria constituted 71.9% of the total bacterial microbiota (including bacterial enteropathogens: *E. coli* 22.2%; *Salmonella* spp. 4.9%; *Yersinia* spp. 3.6%; *Campylobacter* spp. 2.0%). It was also noted that Gram-negative bacteria, including enteropathogenic strains, were present at both hermitized and not or partially hermetized workplaces, especially during the initial stages of technological process, i.e., within wastewater pumping section, screens section, and grit chamber. Like the findings in bioaerosol samples, enteropathogenic species were significantly more frequently isolated from surface swab samples collected at not hermatized workplaces (Chi-square test: *p* = 0.0001, Fisher’s exact test: *p* = 0.0002). All enteropathogenic species isolated from surface swab samples are listed in Table 7.

**Table 7:** Enteropathogenic bacteria identified among surface swab samples with biochemical, MALDI-TOF MS and molecular methods

Workplace	Identification method				
	Biochemical (API)	Spectrometric (MALDI TOF MS)	Molecular (multiplex real-time PCR)		
	Species/genus			Total positive samples	
				NH	H
Wastewater pumping section	*E. coli*	*E. coli*	*E. coli* (EPEC)	4/7	1/3
	*E. coli*	*E. coli*	*E. coli* (ETEC)	2/7	0/3
	*E. coli*	*E. coli*	*E. coli* (OTC)	7/7	1/3
	*Salmonella spp.*	*S. enterica* ssp*. arizonae*	*Salmonella* spp.	2/7	1/3
	*Salmonella* spp*.*	*S. enterica* ssp*. enterica* ser. Hadar	*Salmonella* spp.	2/7	0/3
Screens section	*E. coli*	*E. coli*	*E. coli* (EPEC)	3/4	2/4
	*E. coli*	*E. coli*	*E. coli* (ETEC)	3/4	2/4
	*E. coli*	*E. coli*	*E. coli* (OTC)	4/4	3/4
	*Salmonella* spp.	*Salmonella* spp.	*Salmonella* spp.	3/4	1/4
	*Campylobacter upsaliensis*	*C. upsaliensis*	*Campylobacter spp.*	2/4	1/4
	*Yersinia enterocolitica*	*Y. enterocolitica*	*Y. enterocolitica*	3/4	2/4
Grit chamber	*E. coli*	*E. coli*	*E. coli* (OTC)	3/3	2/7
	*Salmonella* spp.	*Salmonella* spp.	*Salmonella* spp.	1/3	
Bioreactor	*E. coli*	*E. coli*	*E. coli* (OTC)	2/3	1/6
Dewatering sludge section	*E. coli*	*E. coli*	*E. coli* (OTC)	4/4	2/3
Thickening sludge section	*E. coli*	*E. coli*	*E. coli* (OTC)	2/3	1/4

EPEC—enteropathogenic *E. coli*, ETEC—enterotoxigenic *E. coli*, OTC—other than colipathotypes; H—process with hermetization; WH—process without hermetization/ partially hermetized

### Quantitative and qualitative analysis of wastewater samples

**Fig. 5:**
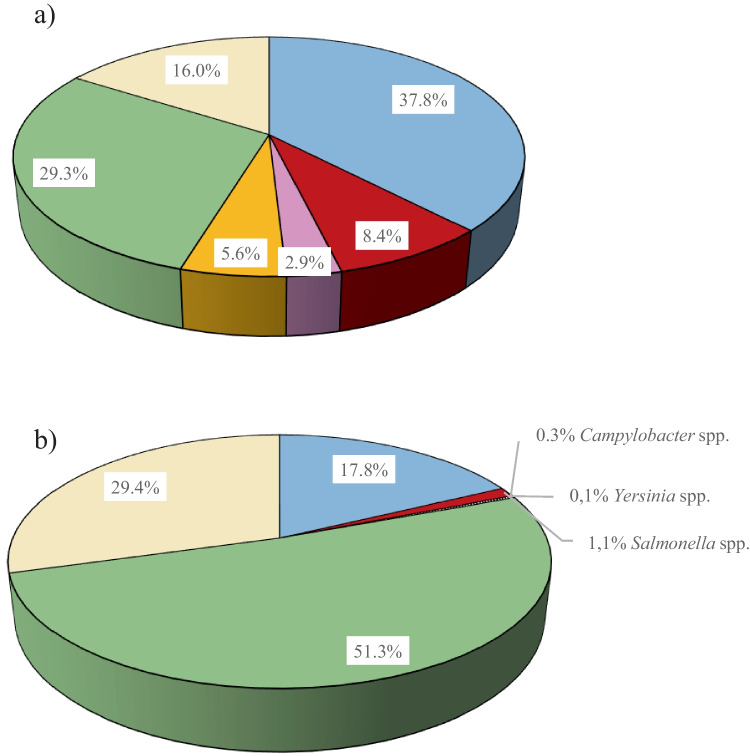
Percentage contribution of bacterial groups to total bacterial microbiota isolated from the untreated (**a**) and treated (**b**) wastewater samples. Notes: *Escherichia coli*, *Salmonella* spp., *Yersinia* spp.,  Campylobacter spp.,  other non-enteropathogenic  Gram-negative rods, Gram-positive bacteria

Average concentrations of bacteria in wastewater samples before treatment ranged from 1.5 × 10^7^ to 4.9 × 10^13^ CFU/mL, while after treatment varied from 5.9 × 10^3^ to 9.7 × 10^5^ CFU/mL. Untreated wastewater contained significantly more bacteria than treated effluents (*p* < 0.05). Percentage distributions of bacterial groups identified in treated and untreated wastewater are shown in Fig. 5. *Escherichia coli* (37.8%), along with non-enteropathogenic Gram-negative rods (29.3%), constituted the predominant group in the untreated wastewater samples, followed by Gram-positive bacteria (16.0%). The species from *Salmonella*, *Campylobacter*, and *Yersinia* genera constituted 8.4%, 5.9%, and 2.9% of the total bacterial microbiota, respectively. In case of treated wastewater samples, non-enteropathogenic Gram-negative rods (51.3%) were the predominant bacterial group, followed by Gram-positive bacteria (29.4%) and *E. coli* (17.8%). *Salmonella* spp. constituted 1.2%, while *Campylobacter* spp. made up to 0.3% of the bacterial microbiota. All enteropathogenic species isolated from wastewater samples are listed in Table 8.

**Table 8:** Enteropathogenic bacteria identified among untreated and treated wastewater samples with biochemical, MALDI-TOF MS, and molecular methods

Identification method		
Biochemical (API)	Spectrometric (MALDI TOF MS)	Molecular (multiplex real-time PCR)
Species/genus		
*E. coli*	*E. coli*	*Escherichia coli* (EPEC)
*E. coli*	*E. coli*	*Escherichia coli* (ETEC)
*E. coli*	*E. coli*	*Escherichia coli* (EHEC)
*E. coli*	*E. coli*	*Escherichia coli* (EIEC)/*Shigella* spp.
*E. coli*	*E. coli*	*E. coli* (OTC)
*Shigella* spp*.*	*Shigella* spp*.*	*Escherichia coli* (EIEC)/*Shigella* spp.
*Salmonella* spp.	*S.* Enteritidis	*Salmonella* spp.
*Salmonella* spp.	*S.* Typhimurium	*Salmonella* spp.
*Salmonella* spp.	*S. enterica* ssp*. arizonae*	*Salmonella* spp.
*Salmonella* spp.	*S. enterica* ssp*. enterica* ser. Hadar	*Salmonella* spp.
*Salmonella* spp.	*S. enterica* ssp*. enterica* ser. Anatum	*Salmonella* spp.
*Salmonella* spp.	*S. enterica* ssp*. enterica* ser. Dublin	*Salmonella* spp.
*Salmonella* spp*.*	*Salmonella* spp.	*Salmonella* spp.
*Yersinia enterocolitica*	*Y. enterocolitica*	*Y. enterocolitica*
*Campylobacter jejuni* spp. *jejuni*	*C. jejuni* spp. *jejuni*	*Campylobacter* spp.
*C. upsaliensis*	*C. upsaliensis*	*Campylobacter* spp.
*C. jejuni* spp*. doylei*	*C. jejuni* spp*. doylei*	*Campylobacter* spp.
*Campylobacter* spp*.*	*Campylobacter* spp*.*	*Campylobacter* spp.

EPEC—enteropathogenic *E. coli*, ETEC—enterotoxigenic *E. coli*, EHEC—enterohemorrhagic *E. coli*, EIEC—enteroinvasive *E. coli*, *Shigella* spp., *Shigella dysenteriae* type 1, OTC—other than colipathotypes

## Discussion

This study confirmed that enteropathogenic bacteria are commonly present at workplaces in wastewater treatment plants. Higher concentrations of total bacteria, including EB, were observed at not or partially hermitized workplaces; however, regardless of the hermetization process in WWTPs, the most microbiologically polluted workplaces, considering both the air and surfaces, were located within the wastewater pumping and screens sections. The bacterial concentrations in the air samples from WWTPs were similar to those obtained by other authors, such as Laitinen et al. (1994), Gotkowska-Płachta et al. (2013), Wang et al. (2018), Yang et al. (2019), and Lou et al. (2021), i.e., varied between 10^1^ and 10^5^ CFU/m^3^. Statistically significant higher concentrations of airborne bacteria were observed at workplaces where the treatment processes were not or only partially hermitized. At all these places, an aeration of wastewater played a crucial role. Aeration of wastewater provides to nonnegligible emission of bioaerosols; however, it is crucial for proper wastewater treatment (Yan et al. 2021). Aeration is utilized to provide oxygen, which promotes the biological process during wastewater treatment but also leads to aerosolization pathogenic bacteria, including Gram-negative enteropathogens. Environmental factors may additionally affect bioaerosol concentration and diversity of bacterial biota. Among them, the source of wastewater treatment, capacity, the type of treatment process, aeration rate, and microclimate parameters play an essential role in determining the concentration of bacteria (Wang et al. 2018). As Michałkiewicz (2018) indicates, bioaerosol emission occurs due to intense flow, transfer, aeration, or turbulence of wastewater and treatment of sludge and storage of screenings and grit. Hermetization of crucial treatment sections at WWTP and fine bubble aeration notably decrease the emission of microorganisms into the air, whereas an intense, turbulent flow of wastewater and lack of ventilation increase the formation of bioaerosols (Fernando and Fedorak 2005). According to Dehghani et al. (2018) and Gotkowska-Płachta et al. (2013), both temperature and humidity of the air may positively correlate with culturable bioaerosol concentration; however, in our study, neither relative air humidity nor temperature significantly influenced bacterial aerosol concentrations (*p* > 0.05).

Our study indicated also that despite of hermitization of wastewater tyreatment processes, Gram-negative EB were present at all workplaces. In case of hermetizated WWTP areas, Gram-negative bacteria constituted below 36% of total airborne bacteria (including 4.9% of EB), while within not or partially hermetizated WWTP sections they formed over 75% (including 24.6% enteropathogenic bacteria). As the main source of Gram-negative bacteria is untreated wastewater, the highest concentration of bacteria was noted within the mechanical wastewater treatment sections (Gotkowska-Płachta et al. 2013). Such abundance of Gram-negative bacteria in the air, including *Enterobacteriaceae* strains (*E. coli, Salmonella* spp.), may be a result of high air humidity (52.7–60.3%) within tested areas, which favors the occurence of Gram-negative strains in the air. Similar observation was noticed by Gotkowska-Płachta et al. (2013) that high air humidity (60.5 ± 18.6%) significantly positively correlated to the level of *Enterobacteriaceae* in the air samples. According to available data, the percentage of Gram-negative bacteria in bioaerosol samples from WWTPs ranged between 35 and 55% of total airborne bacteria, and a large part of them formed *Enterobacteriaceae*, including *Salmonella* spp., *Y. enterocolitica*, and *E. coli* (Dehghani et al. 2018; Wlazło et al. 2002). Although *Enterobacteriaceae* are strictly related to the aquatic environment, the prevalence of *Salmonella* spp. and *E. coli*, including pathotypes (ETEC) in bioaerosols, was already proved in several studies (Gerba et al. 2008; Wu et al. 2011; Farling et al. 2019; Li et al. 2019).

Untreated wastewater and screenings, sludge, and grit residues derived from it may contaminate workplace surfaces in WWTPs. Such polluted surfaces may become a secondary emission source for bioaerosols. Deposited microbial particulates can also be resuspended by air movement caused by wind, ventilation, or human activities at the workplace (such as walking or opening doors or windows (Inizan 2018; Alsved et al. 2020). This study confirmed that surfaces of machine handles and valves, handrails, and conveyor belts in WWTPs may be contaminated with EB. Consequently, bacterial enteropathogens may be transmitted via contaminated workars’ hands and lead to fecal–oral infections (Svenungsson et al. 2000). Moreover, dermal contact with some strains of *E. coli* and *Salmonella* spp. may play an important role in skin irritation and cutaneous infection among exposed workers (Marzano et al. 2003; Petkovšek et al. 2009).

The state of knowledge about biological risks in work environments is still relatively incomplete (Santos et al. 2020). Although a few hundred million workers around the world are exposed to airborne biological agents, and in the EU, biohazard prevention is mandatory, there are still no widely accepted threshold limit values for bacterial contaminants. The Polish Expert Group for Biological Agents of the Interdepartmental Commission for Maximum Admissible Concentrations and Intensities for Agents Harmful to Health in the Working Environment at the Central Institute for Labour Protection–National Research Institute (CIOP–PIB), taking into account ‘environmental factors’, proposed the threshold limit values (TLV) for microbiological agents in the air of occupational and non-occupational environments (Table 9) (Pośniak and Skowroń 2022). The concentrations of total bacteria, as well as Gram-negative bacteria, in the air at all workplaces in WWTPs were below the proposed TLVs for workplaces polluted with organic dust. However, the presence of bacterial enteropathogens indicates that risk assessment methods for occupational exposure should also take into account the results of qualitative analysis (Table 9).

**Table 9:** Threshold limit values for bioaerosols proposed by Polish Expert Group for Biological Agents of the Interdepartmental Commission for Maximum Admissible Concentrations and Intensities for Agents Harmful to Health in the Working Environment

Microbial agent	Workplaces polluted with organic dust
Mesophilic bacteria	100,000 CFU/m^3^
Gram-negative bacteria	20,000 CFU/m^3^
Microbial agents from risk groups 3 and 4	0 CFU/m^3^

Increased risk for enteric infections is associated with workplace exposures to enteropathogens (Su et al. 2017; Duijster et al. 2019), and this study also underlines the necessity of precise control of wastewater treatment plant workers’ exposure to EB, especially in not hermitized sections of WWTPs where mechanical agitation or forced aeration of wastewater takes place (Heinonen-Tanski et al. 2009). The existence of this type of biothreats causes a need for introduction of analytical strategy for the fast and accurate detection and identification of enteropathogenic bacteria in the occupational environment of WWTPs.

Traditional culture-based methods for pathogen detection are usually time-consuming and require additional approval techniques (Bursle and Robson 2016). Moreover, while conventional bacterial testing methods, relying on selective and chromogenic media, are commonly used for detection and identification of both indicator and pathogenic microorganisms, the use of molecular methods for this purpose has been increasing due to their numerous practical benefits (Salmonova and Bunesova 2017). The specificity, sensitivity, and reduced processing time of molecular techniques make them suitable for aerobiological and surface monitoring, particularly for detecting small numbers of targeted microorganisms (Alvarez et al. 1995). A reliable alternative for culture-based detection methods of microorganisms in environmental samples is the PCR assay. So far, molecular screening of wastewater has been carried out for the rapid detection of multiple gastrointestinal pathogens in biological waste (Ørmen and Madslien 2018). Multiplex real-time PCR assays allow accurate detection of nucleic acids to be extended to pathogenic bacteria, including EB in environmental samples (Ørmen et al. 2019).

Advantages and disadvantages of all methods used in this study to identify bacterial biota are listed in Table 10. This study confirmed that commercially available multiplex real-time PCR kits are suitable for fast detection and identification of enteropathogenic bacteria in occupational environment. Although, in some cases, they may not identify pathogens to the species or subspecies levels, usually recognition to the genus level is sufficient for exposure assessment. Such evaluation requires the classification of a given biological agent into one of four risk groups based on its level of pathogenicity, virulence, transmission, and availability of effective prophylactic measures and treatment. This classification, however, depends on the premises followed by a given organization that established or recognize it, such as the World Health Organization, Health Canada, European Union (EU), or European Federation of Biotechnology (ISC schemes 2023). Multiplex real-time PCR may also be suitable for assessing the efficiency of mechanical, biological, chemical, and combined wastewater treatment processes in removing pathogenic bacteria (Osińska et al. 2018). However, even advanced treatment methods including biological and physicochemical processes, as indicated by Ørmen et al. (2019), do not eliminate all pathogens from water. Pathogenic bacteria like *Campylobacter* spp., *Salmonella* spp., *Y. enterocolitica*, enteroaggregative *E. coli*, enteropathogenic *E. coli*, enterotoxigenic *E. coli*, shigatoxin-positive *E. coli*, *E. coli* O157:H7, *V. cholera*e, and *Shigella* spp. were detected in treated wastewater subjected to mechanical, chemical, and biological treatment methods (Toze 1997). These pathogens are considered a serious public health problem because their presence in the environment can result in numerous diseases in the general population (Kulinkina et al. 2016; Ashbolt 2015). The results of this study confirmed that wastewater effluents may contain bacterial enteropathogens, such as *E. coli* (EPEC, ETEC), and strains of *Salmonella* and *Campylobacter* genera. If the discharged treated wastewater is reused as drinking or recreational water or is used for irrigation, it may again become a source of contamination with enteropathogenic bacteria, posing a risk to human health (Su et al. 2017; Duijster et al. 2019). It should be considered as probable scenario that some *E. coli* strains with uropathogenic properties, which survived treatment stages applied in WWTPs, may be again released into the environment (Anastasi et al. 2012).

**Table 10:** Advantages and disadvantages of bacterial enteropathogens detection and identification methods

Detection/identification method	Advantages	Disadvantages
Culture-based	- no need of special equipment	- assess culturable part of microbiota only - time-consuming
	- sensitive method	- necessity of broad range of proper media
	- rather inexpensive (excluding chromogenic media)	- choosing of typical colonies is subjective
	- may provide additional information such as antibiotic resistance, nutrient and growth requirements	- the risk of omitting the colonies of enteropathogenic bacteria
		- limitations resulting from heterogeneity of samples matrices (bacteria are not uniformly distributed in environment or in samples), incomplete selectivity of culture medium, growth conditions, and different growth rate of microorganisms and strong influence of companion microbiota
		- identification approval with other methods is reqiured
Biochemical (API)	- biochemical kits are commercially available (API)	- this method selects microorganisms capable of growing under the experimental conditions, favors fast growing microorganisms
	- provide the identification percentage (probability of species identification)	- high sensitivity to inoculum density
	- may provide additional information such as antibiotic resistance, nutrient and growth requirements	- reflects the potential, rather than in situ, metabolic diversity
		- carbon sources used for the tests may not correspond to those present in the sample
		- the database of identified species is limited
Spectrometric (MALDI-TOF MS)	- rapid (results within 4 h)	- needs special, technically advanced and expensive equipment
	- cost per isolate is lower than in biochemical method	- single colony from primary culture plates is required for analysis
	- a wide database of identified species	
	- in some cases (*Salmonella* spp.) more specific than biochemical method	
Molecular (multiplex real-time PCR)	- ready to use kits are available	- possible inhibition of PCR by co-extracted contaminants
	- rapid (results within 1.5 h)	- needs special, technically advanced and expensive equipment
	- high sensitivity and specificity	- in some cases identification to genus level only (e.g., while using commercial available kits)
	- relatively inexpensive	
	- no need of single colony from primary culture plates is required for analysis (pool samples)	
	- able to identify and detect several pathogens in one reaction (multiplex approach)	

## Conclusions

This study revealed that the WWTP environment contains high amount of bacteria including enteropathogenic bacteria strains, which can be found in sewage, in the air (in form of bioaerosols), and on surfaces. Thus, it may significantly influence the health status of WWTPs workers. The control of enteropathogenic bacterial air and surface contamination, utilizing rapid PCR-based methods, should be routinely carried out as a part of hygienic quality assessment within WWTPs. Moreover, identification and classification of isolated microorganisms to proper risk group should be an immanent part of occupational risk assessment.

In conclusion, the assessment and characterization of bacterial enteropathogens play a pivotal role in establishing a scientific foundation for prevention through exposure reduction, particularly in WWTPs. The introduction and enhancement of appropriate hygienic practices, encompassing measures like hand washing, thorough cleaning, and disinfection procedures, and the implementation of hermetization strategies, directly contribute to elevating the microbial quality of the processing environment. This improvement, in turn, enhances the overall safety of WWTPs.

## References

[CR1] Albatanony MA, El-Shafie MK (2011). Work-related health effects among wastewater treatment plants workers. Int J Occup Environ Med.

[CR2] Alsved M, Bourouiba L, Duchaine C, Löndahl J, Marr LC, Parker ST, Prussin AJ, Thomas RJ (2020). Natural sources and experimental generation of bioaerosols: challenges and perspectives. Aerosol Sci Technol.

[CR3] Alvarez AJ, Buttner MP, Stetzenbach LD (1995). PCR for bioaerosol monitoring: sensitivity and environmental interference. Appl Environ Microbiol.

[CR4] Anastasi EM, Matthews B, Stratton HM, Katouli M (2012). Pathogenic Escherichia coli found in sewage treatment plants and environmental waters. Appl Environ Microbiol.

[CR5] APHA (2001). Standard Methods for the Examination of Water and Wastewater.

[CR6] Ashbolt NJ (2015). Microbial contamination of drinking water and human health from community water systems. Curr Environ Health Rep.

[CR7] Bursle E, Robson J (2016). Non-culture methods for detecting infection. Aust Prescr.

[CR8] Chahal C, van den Akker B, Young F, Franco C, Blackbeard J, Monis P (2016). Pathogen and particle associations in wastewater: significance and implications for treatment and disinfection processes. Adv Appl Microbiol.

[CR9] Dehghani M, Sorooshian A, Ghorbani M, Fazlzadeh M, Miri M, Badiee P, Parvizi A, Ansari M, Baghani AN, Delikhoon M (2018). Seasonal variation in culturable bioaerosols in a wastewater treatment plant. Aerosol Air Qual Res.

[CR10] Duijster JW, Franz E, Neefjes JJC, Mughini-Gras L (2019). Occupational risk of salmonellosis and campylobacteriosis: a nationwide population-based registry study. Occup Environ Med.

[CR11] EPA Environmental Protection Agency, Exposure pathways to high-consequence pathogens in the wastewater collection and treatment systems office, EPA/600/R-18/221, 2018, www.epa.gov/homeland-security-research

[CR12] European Committee for Standardization (CEN). Workplace atmosphere—guidelines for measurement of airborne microorganisms and endotoxin (Standard No. EN 13098:2019). Brussels, Belgium: CEN; 2019.

[CR13] Farling S, Rogers T, Knee JS, Tilley EA, Brown J, Deshusses MA (2019). Bioaerosol emissions associated with pit latrine emptying operations. Sci Total Environ.

[CR14] Fernando NL, Fedorak PM (2005). Changes at an activated sludge sewage treatment plant alter the numbers of airborne aerobic microorganisms. Water Res.

[CR15] Friis L, Agréus L, Edling C (1998). Abdominal symptoms among sewage workers. Occup Med (lond).

[CR16] Gerba CP, Castro-del Campo N, Brooks JP, Pepper IL (2008). Exposure and risk assessment of Salmonella in recycled residuals. Water Sci Technol.

[CR17] Gotkowska-Płachta A, Filipkowska Z, Korzeniewska E, Janczukowicz W, Dixon B, Gołaś I, Szwalgin D (2013). Airborne microorganisms emitted from wastewater treatment plant treating domestic wastewater and meat processing industry wastes. Clean - Soil Air Water.

[CR18] Grisoli P, Rodolfi M, Villani S, Grignan E, Cottica D (2009) Assessment of airborne microorganism contamination in an industrial area characterized by an open composting facility and a wastewater treatment plant. Environ Res 109(2):135–142. 10.1016/j.envres.2008.11.001 10.1016/j.envres.2008.11.001 19131053

[CR19] Han Y, Li L, Liu J (2013). Characterization of the airborne bacteria community at different distances from the rotating brushes in a wastewater treatment plant by 16S rRNA gene clone libraries. J Environ Sci.

[CR20] Heinonen-Tanski H, Reponen T, Koivunen J (2009). Airborne enteric coliphages and bacteria in sewage treatment plants. Water Res.

[CR21] Inizan M (2018) Turbulence-particle interactions on surfaces. SM Thesis, Massachusetts Institute of Technology, USA

[CR22] ISC schemes (2023) International classifications schemes for micro-organisms based on their biological risks. https://www.biosafety.be/content/contained-use-international-classifications-schemes-micro-organisms-based-their-biological

[CR23] Jabeen R, Ahmed ME, Hamouda MA, Aly Hassan A (2023). Bioaerosols in wastewater treatment plants: trends, recent advances, and the influence of SARS-CoV-2 outbreak. Water.

[CR24] Jeggli S, Steiner D, Koller H (2004). Hepatitis E, Helicopacter pylori and gastrointestinal symptoms in workers exposed to waste water. Occup Environ Med.

[CR25] Jia S, Zhang X (2020) Biological HRPs in wastewater. High-Risk Pollutants in Wastewater 41–78. 10.1016/B978-0-12-816448-8.00003-4

[CR26] Jones K (2001). Campylobacters in water, sewage and the environment. J Appl Microbiol.

[CR27] Kozdrój J, Frączek K, Ropek D (2019). Assessment of bioaerosols in indoor air of glasshouses located in a botanical garden. Building Environ.

[CR28] Kulinkina A, Mohan V, Francis M (2016). Seasonality of water quality and diarrheal disease counts in urban and rural settings in south India. Sci Rep.

[CR29] Laitinen S, Kangas J, Kotimaa M, Liesivuori J, Martikainen PJ, Nevalainen A, Sarantila R, Husman K (1994). Workers exposure to airborne bacteria and endotoxins at industrial wastewater treatment plants. Am Ind Hyg Assoc J.

[CR30] Langeland G (1982). Salmonella spp. in the working environment of sewage treatment plants in Oslo Norway. Appl Env Microbiol.

[CR31] Li Z, Wang H, Zheng W, Li B, Wei Y, Zeng J, Lei C (2019). A tracing method of airborne bacteria transmission across built environments. Build Environ.

[CR32] Lou M, Liu S, Gu C, Hu H, Tang Z, Zhang Y, Xu C, Li F (2021). The bioaerosols emitted from toilet and wastewater treatment plant: a literature review. Environ Sci Pollut Res Int.

[CR33] Marzano AV, Mercogliano M, Borghi A, Facchetti M, Caputo R (2003). Cutaneous infection caused by Salmonella typhi. J Eur Acad Dermatol Venereol.

[CR34] Michałkiewicz M (2018). Comparison of wastewater treatment plants based on the emissions of microbiological contaminants. Environ Monit Assess.

[CR35] Muzaini K, Yasin SM, Ismail Z, Ishak AR (2021). Systematic review of potential occupational respiratory hazards exposure among sewage workers. Front Public Health.

[CR36] Ørmen Ø, Madslien EH (2018). Molecular monitoring of enteropathogens in sewage during NATO exercise trident juncture 2018: potential tool in early outbreak warning?. Military Med.

[CR37] Ørmen Ø, Aalberg K, Madslien EH (2019). Multiplex polymerase chain reaction detection of enteropathogens in sewage in Norway. Acta Vet Scand.

[CR38] Osińska A, Korzeniewska E, Harnisz M, Niestępski S (2018) The prevalence of virulence genes specific for Escherichia coli in wastewater samples from wastewater treatment plants with the activated sludge process, E3S Web Conf 44:00133. 10.1051/e3sconf/20184400133

[CR39] Petkovšek Ž, Eleršič K, Gubina M, Žgur-Bertok D, Starčič Erjavec M (2009). Virulence potential of Escherichia coli isolates from skin and soft tissue infections. J Clinical Microbiol.

[CR40] Pośniak M, Skowroń J (2022) Harmful agents in the working environment. Limit values 2022. CIOP-PIB, Warsaw (in Polish)

[CR41] Salmonova H, Bunesova VN (2017). Methods of studying diversity of bacterial comunities: a review. Sci Agricultur Bohem.

[CR42] Santos J, Ramos C, Vaz-Velho M, Vasconcelos Pinto M (2020) Occupational exposure to biological agents. In: Arezes, P., Boring, R. (eds) Advances in safety management and human performance. AHFE 2020. Advances in Intelligent Systems and Computing, vol. 1204. Springer, Cham. 10.1007/978-3-030-50946-0_9

[CR43] Su CP, de Perio MA, Fagan K, Smith ML, Salehi E, Levine S, Gruszynski K, Luckhaupt SE (2017). Occupational distribution of Campylobacteriosis and Salmonellosis cases—Maryland, Ohio, and Virginia, 2014. MMWR Morb Mortal Wkly Rep.

[CR44] Suzuki Y, Niina K, Matsuwaki T, Nukazawa K, Iguchi A (2018). Bacterial flora analysis of coliforms in sewage, river water, and ground water using MALDI-TOF mass spectrometry. J Environ Sci Health A Tox Hazard Subst Environ Eng.

[CR45] Svenungsson B, Lagergren A, Ekwall E, Evengård B, Hedlund KO, Kärnell A, Löfdahl S, Svensson L, Weintraub A (2000). Enteropathogens in adult patients with diarrhea and healthy control subjects: a 1-year prospective study in a Swedish clinic for infectious diseases. Clin Infect Dis.

[CR46] Teklehaimanot GZ, Genthe B, Kamika I, Momba MN (2015). Prevalence of enteropathogenic bacteria in treated effluents and receiving water bodies and their potential health risks. Sci Total Environ.

[CR47] Toze S (1997). Microbial pathogens in wastewater. Literature review for urban water systems multi-divisional research program. Technical report no 1/97 CSIRO, 1997, Australia

[CR48] Wang Y, Lan H, Li L (2018). Chemicals and microbes in bioaerosols from reaction tanks of six wastewater treatment plants: survival factors, generation sources, and mechanisms. Sci Rep.

[CR49] Wlazło A, Pastuszka JS, Łudzeń-Izbińska B (2002). Assessment of workers' exposure to airborne bacteria at a small wastewater treatment plant. Med Pr.

[CR50] Wu MJ, Feng YS, Sung WP, Surampalli RY (2011). Quantification and analysis of airborne bacterial characteristics in a nursing careinstitution. J Air Waste Manag Assoc.

[CR51] Wu L, Ning D, Zhang B (2019). Global diversity and biogeography of bacterial communities in wastewater treatment plants. Nat Microbiol.

[CR52] Yan C, Gui ZC, Wu JT (2021) Quantitative microbial risk assessment of bioaerosols in a wastewater treatment plant by using two aeration modes. Environ Sci Pollut Res Int 28(7):8140–8150. 10.1007/s11356-020-11180-6 10.1007/s11356-020-11180-6 33051848

[CR53] Yang K-X, Li L, Wang Y-J, Xue S, Han Y-P, Liu JX (2019). Airborne bacteria in a wastewater treatment plant: emission characterization, source analysis and health risk assessment. Water Res.

[CR54] Yi J, Shane AL (2018) Approach to the diagnosis and management of gastrointestinal tract infections, Editor(s): Long SS, Prober CG, Fischer M, Principles and practice of pediatric infectious diseases (fifth edition), Elsevier, 376–383

